# Shear Tests on Polyurethane Flexible Joints

**DOI:** 10.3390/ma19010097

**Published:** 2025-12-26

**Authors:** Łukasz Hojdys, Piotr Krajewski, Arkadiusz Kwiecień

**Affiliations:** 1 Faculty of Civil Engineering, Cracow University of Technology, Warszawska 24, 31-155 Krakow, Poland; piotr.krajewski@pk.edu.pl; 2 FlexAndRobust Systems Ltd., Warszawska 24, 31-155 Krakow, Poland; ak@flexandrobust.com

**Keywords:** flexible joint, flexible polyurethane, initial shear strength, masonry, concrete block

## Abstract

This paper investigates the behavior of PM-type polyurethane flexible joints connecting structural components. Although flexible polyurethanes are known for their energy dissipation capacity and ability to accommodate large deformations—particularly under seismic actions—research addressing their performance under shear loading remains limited. The primary objective of this work was to characterize these joints under varying levels of normal stress, identify failure modes, and estimate key mechanical parameters. Nine masonry triplet specimens, composed of concrete units and PM-type polyurethane, were subjected to shear testing using a procedure adapted from EN 1052-3. Tests were carried out at three precompression levels: 0.2, 0.6, and 1.0 N/mm^2^. Tensile tests were further performed to calibrate material models. The results showed that increasing precompression led to higher ultimate shear loads. All specimens failed due to shear failure at the unit–flexible joint interface, with no damage observed in the masonry units. Based on linear regression following EN 1052-3, the initial shear strength was determined to be 0.729 N/mm^2^, corresponding to a friction coefficient of 0.14.

## 1. Introduction

MEZeroE is a European research and innovation initiative funded under the Horizon 2020 programme, focused on advancing building envelope technologies for nearly zero-energy buildings (nZEBs). The project establishes a distributed open-innovation ecosystem that connects manufacturers, research institutions, and laboratories via a virtual testing and validation platform. This ecosystem includes nine measurement and verification lines (PM&VL) as well as services supporting the commercialization of innovative solutions, facilitating faster introduction of sustainable building technologies to the market. By providing access to state-of-the-art experimental infrastructure and promoting collaboration among stakeholders, MEZeroE enables systematic evaluation and optimization of new materials and construction methods, aligning research outcomes with industry needs [1].

Within the MEZeroE framework, researchers at Cracow University of Technology investigated polyurethane-based materials for applications in building envelopes. The studied products are designed for connecting structural components, strengthening, repairing damaged elements, and crack injections. As part of the pilot measurement and verification line (PM&VL7), a comprehensive experimental program was implemented to assess the fundamental mechanical, thermal, and acoustic properties of the flexible polyurethanes. The results are intended to support both performance validation and market introduction of the product, contributing to the broader adoption of durable and sustainable building envelope solutions [2].

**Figure 1:**
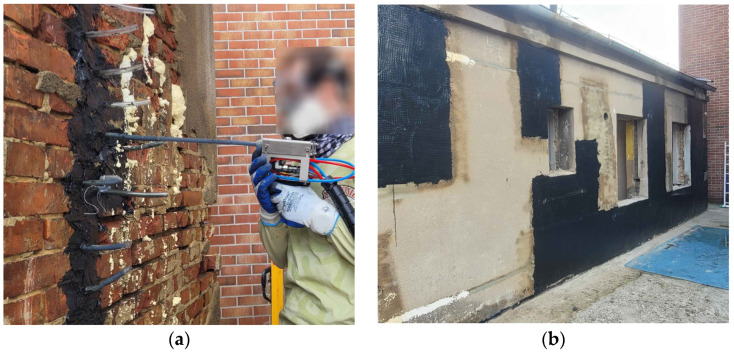
Repair of cracked masonry walls: (**a**) practical application of a flexible polyurethane joint for consolidating cracked masonry walls; (**b**) walls repaired with fiber-reinforced polyurethane strengthening system.

The flexible polyurethanes are used in surface strengthening systems for structural elements. In these systems, meshes made of steel, glass, or carbon fibers are embedded in a flexible matrix [3,4]. Polyurethane enables energy absorption and dissipation and accommodates large deformations, which is particularly advantageous when structures are exposed to seismic actions. The flexible matrix reduces stress concentrations transferred from the stiff fibers to the brittle and weak masonry units—brick, stone, or concrete [3]. The benefits of this solution have been demonstrated in shake table tests [5], push-over tests [6], and forced harmonic vibration tests [7]. In addition, polyurethane-based flexible systems exhibit thermal stability [8], resistance to environmental effects [4], and reversibility [9]. Flexible polyurethanes can also be used for connecting structural components [10,11,12]. This type of connection is commonly referred to in the literature as “flexible joint” [12,13,14,15,16]. They have been applied to the repair of cracked concrete elements subjected to thermal and bending effects [17,18], to the connection of masonry infill walls within reinforced-concrete frames [5,6], and to the joining of precast reinforced-concrete elements [19,20]. Within the MezeroE programme, this material has also been tested for the injection repair of damaged masonry in the building designated as the “Living Laboratory” (Figure 1).

The design of connections between structural elements using flexible joints requires a thorough understanding of the behavior of the material under loading. Equally important is the interaction between the flexible joint and the connected components. The available literature on flexible joints in civil engineering focuses on determining the mechanical parameters of polyurethanes under uniaxial tensile loading [20], on bond tests of the strengthening layer to the substrate [3,4,21], and on bending tests of beams [11,16]. However, there is a lack of research addressing the behavior of flexible joints under shear loading, including complex stress states. To determine the mechanical characteristics of a flexible joint subjected to shear, the test procedure for initial shear strength specified in EN 1052-3 was adapted [22]. This method is the standard approach for assessing the properties of masonry under shear loading acting parallel to the bed joints [23,24,25,26,27,28,29,30,31].

This paper presents the results of initial shear strength tests performed using PM-type polyurethane and concrete masonry units. The main objective of the study was to investigate the behavior of such joints under shear loading at various levels of normal stress (precompression), to identify the failure modes, and to estimate the mechanical parameters governing their shear response.

## 2. Materials and Methods

### 2.1. Materials

**Figure 2:**
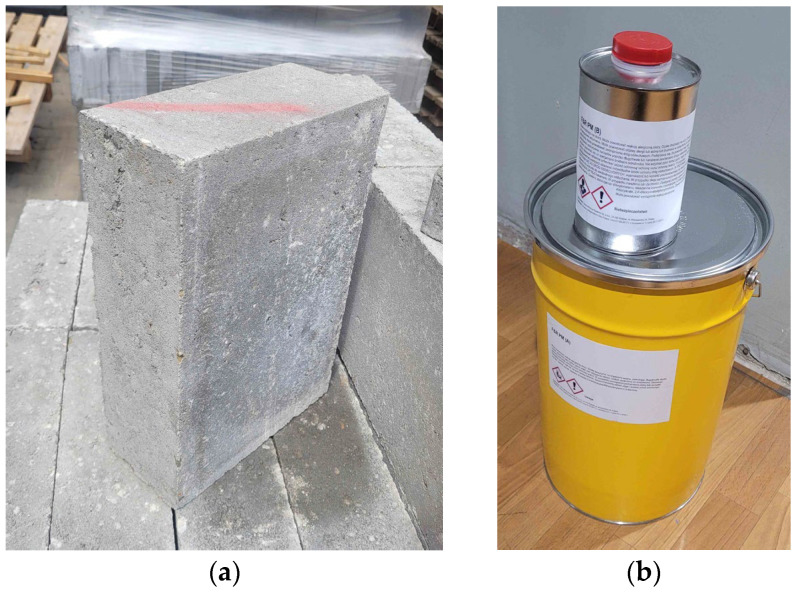
Materials used in the research: (**a**) concrete masonry unit; (**b**) the components of highly deformable polyurethane of PM-type.

Initial shear strength tests were carried out on masonry specimens made with concrete masonry units connected by a flexible joint. The concrete blocks used in the tests had dimensions of 380 × 250 × 120 mm^3^ (Figure 2a). Based on the manufacturer’s declaration prepared according to EN 771-3 [32], the mean compressive strength and bulk density of these masonry elements were 15 N/mm^2^ and 2200 kg/m^3^, respectively.

The flexible joint was made of a two-component, highly deformable polyurethane of PM-type (Figure 2b). The results of uniaxial tensile tests of this material, performed in accordance with ISO 527-1 [33], are presented, among others, in [11,20] and in the technical data sheet provided by the manufacturer. According to these sources, the PM-type polyurethane exhibits the following mechanical properties: tensile strength up to 1.6 N/mm^2^ ultimate strain up to 130%, and a Young’s modulus of approximately 4.2 N/mm^2^.

Additionally, ref. [11] presented results of elastic modulus tests conducted on PM-type flexible joint samples of various geometries under uniaxial compression, tension, and bending. Depending on the specimen geometry and loading mode, the obtained elastic modulus values ranged from 6.9 N/mm^2^ to 11.8 N/mm^2^ in tension and from 2.2 N/mm^2^ to 6.0 N/mm^2^ in compression.

**Figure 3:**
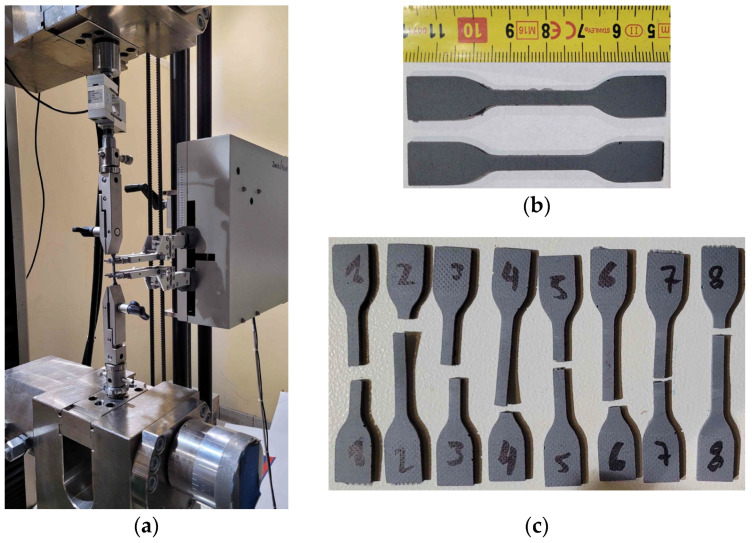
Determination of mechanical properties of polyurethane of PM-type: (**a**) test setup for tensile test; (**b**) dumb-bell specimens of type-2; (**c**) failure modes of tested specimens.

**Figure 4:**
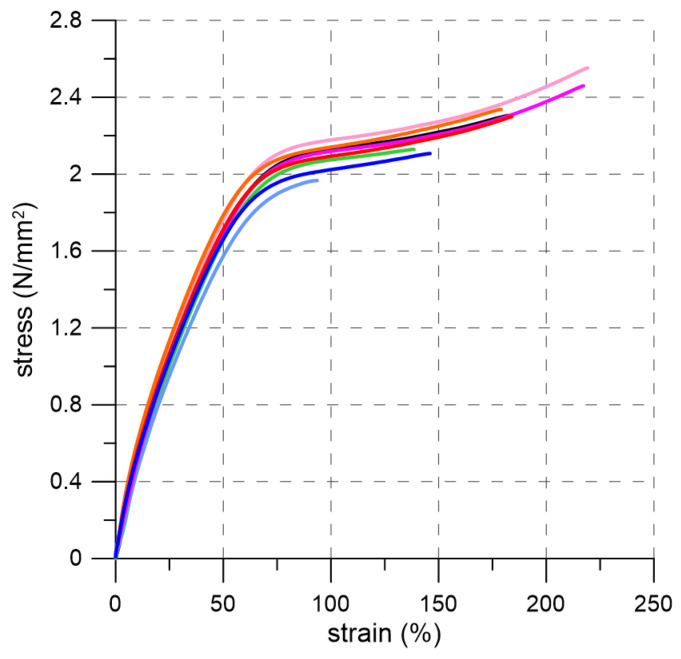
Stress–strain curves of tensile test for PM-type polyurethane.

Since this polyurethane behaves as an elastomer it was decided to perform additional mechanical tests in accordance with ISO 37 [34]. The standard specifies the methods for determining the tensile stress–strain properties of rubber-like materials. The tests were conducted on eight dumb-bell specimens of type-2 (Figure 3) using ZwickRoell Z100 (ZwickRoell, Ulm, Germany) testing machine equipped with HBM SM100 load cell and an extensometer. The gauge length was 20 mm. The tests were performed under displacement control at a rate of 500 mm/min. A view of the test setup and the failure modes of the specimens are shown in Figure 3. Based on these tests, the following mechanical parameters were determined for PM-type polyurethane: tensile strength of 2.27 N/mm^2^ and elongation at break of 170%. Additionally, the secant modulus of elasticity was estimated as 6.22 N/mm^2^ and 5.22 N/mm^2^ for strain intervals of 0–2% and 0–10%, respectively. The stress–strain curves obtained from the tests are presented in Figure 4.

### 2.2. Specimen Preparation

**Figure 5:**
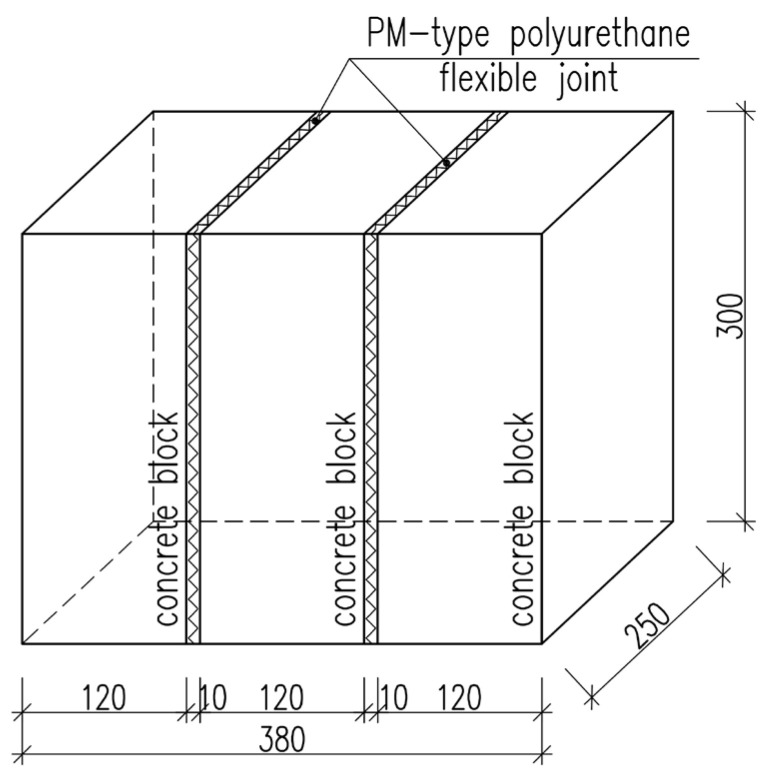
Geometry of shear test specimen.

In order to determine shear behavior of the highly deformable polyurethane of the PM type used as a flexible joint, specimens of the geometry shown in Figure 5 were prepared. The specimen geometry was selected based on the EN 1052-3 standard [22]. Considering the dimensions of the masonry element, a Type A specimen geometry was adopted.

**Figure 6:**
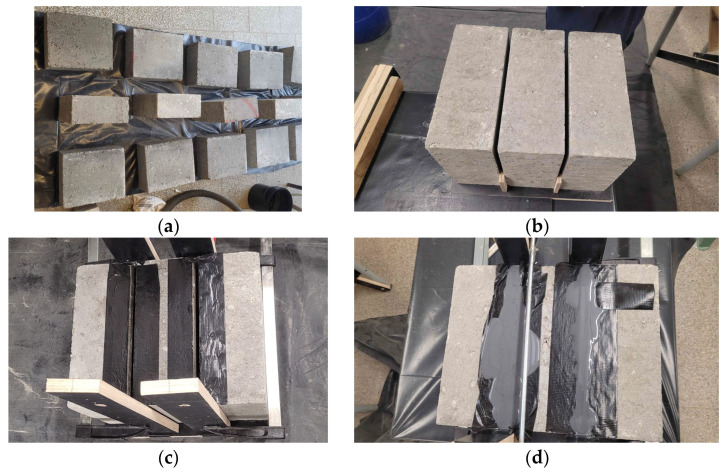

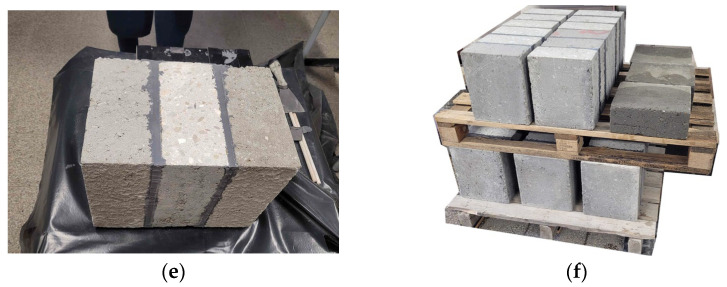
Stages of specimen preparation: (**a**) masonry units cut to dimensions of 300 × 250 × 120 mm^3^; (**b**) stabilization of the blocks; (**c**) sealing of the gaps; (**d**) specimen after application of PM-type polyurethane; (**e**) view of a typical test specimen; (**f**) specimens ready for testing.

The specimens were prepared through six main steps. First, the masonry units were cut to dimensions of 300 × 250 × 120 mm. The surfaces of the concrete blocks were then cleaned with compressed air to remove any dust. Next, a single-component polyurethane primer was applied to improve adhesion between the flexible joints and the masonry units. Three concrete blocks were arranged to create 10 mm wide gaps between their bed surfaces, and the lateral surfaces of the specimen were protected to prevent polyurethane leakage. Finally, the gaps between the bed surfaces were filled with PM-type polyurethane (Figure 6). Following this procedure, nine test specimens were produced, each measuring 380 × 300 × 120 mm^3^, with bed joints 10 mm thick (Figure 6e).

### 2.3. Test Setup and Test Procedure

**Figure 7:**
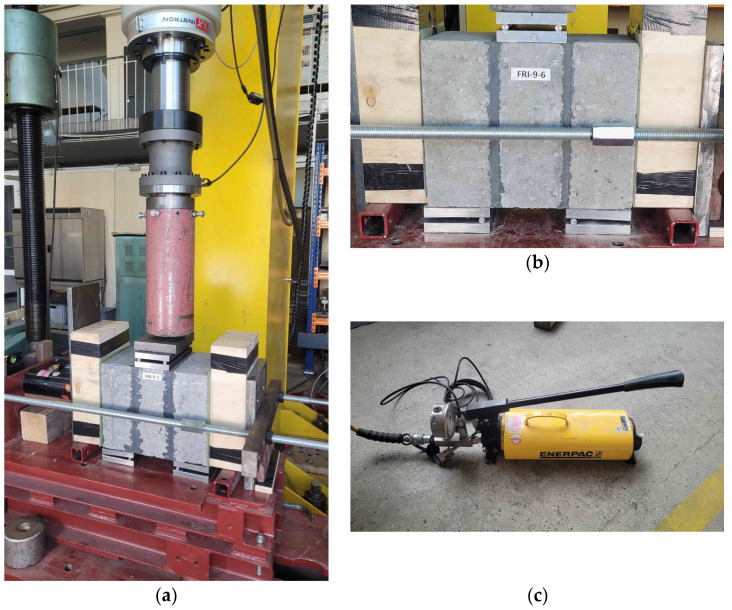
Test setup: (**a**) front view of the setup; (**b**) detailed view of a specimen during testing; (**c**) hydraulic hand pump used to apply precompression.

The specimens were tested in shear under four-point loading configuration, with precompression applied perpendicular to the bed joints, in accordance with EN 1052-3 [22]. Although this standard refers to testing masonry with joints made of mineral mortar, it was adapted in the present study due to the lack of appropriate standards for testing flexible joints. The specimens were positioned on the test setup such that the outer masonry units were supported, while the load was applied to the central masonry unit. Roller bearings, consisting of 16 mm thick steel plates and 12 mm diameter steel rods, were used. Loading beams and a hydraulic actuator were applied to the ends of the test specimen to generate the precompression, with the actuator controlled manually using a hand pump (Figure 7c). In the first stage of testing, the specimens were subjected to precompression, followed by vertical loading applied under displacement control at a rate of 2 mm/min. The vertical load was applied using an Instron 250 kN hydraulic actuator (Instron, Darmstadt, Germany) until a vertical displacement of approximately 25–30 mm was reached. During testing, the applied vertical load and actuator displacement were recorded, and the precompression level was monitored. Additionally, the digital image correlation (DIC) method was used to determine shear strain.

Three specimens were tested at each of three precompression levels. Specimens FRI-9-3, FRI-9-4, and FRI-9-5 were tested under a precompression of 0.2 N/mm^2^, specimens FRI-9-1, FRI-9-6, and FRI-9-7 under 0.6 N/mm^2^, and specimens FRI-9-2, FRI-9-8, and FRI-9-9 under 1.0 N/mm^2^ (Table 1).

A general view of the test setup and a detailed view of a specimen with roller bearings are shown in Figure 7.

## 3. Results

The results of the shear tests performed on masonry units with flexible joints are summarized in Table 1, including peak load (F_max_), vertical displacement at peak load (δ_Fmax_), and the corresponding failure mode. The failure modes are classified according to the schemes presented in EN 1052-3 [22]. For the purposes of the present study, the failure modes were adapted to account for the replacement of the mineral mortar with a flexible joint. The following failure modes were considered: FM1—shear failure in the unit/flexible joint bond area; FM2—shear failure only in the flexible joint; FM3—shear failure in the unit; and FM4—crushing and/or splitting failure in the units.

**Figure 8:**
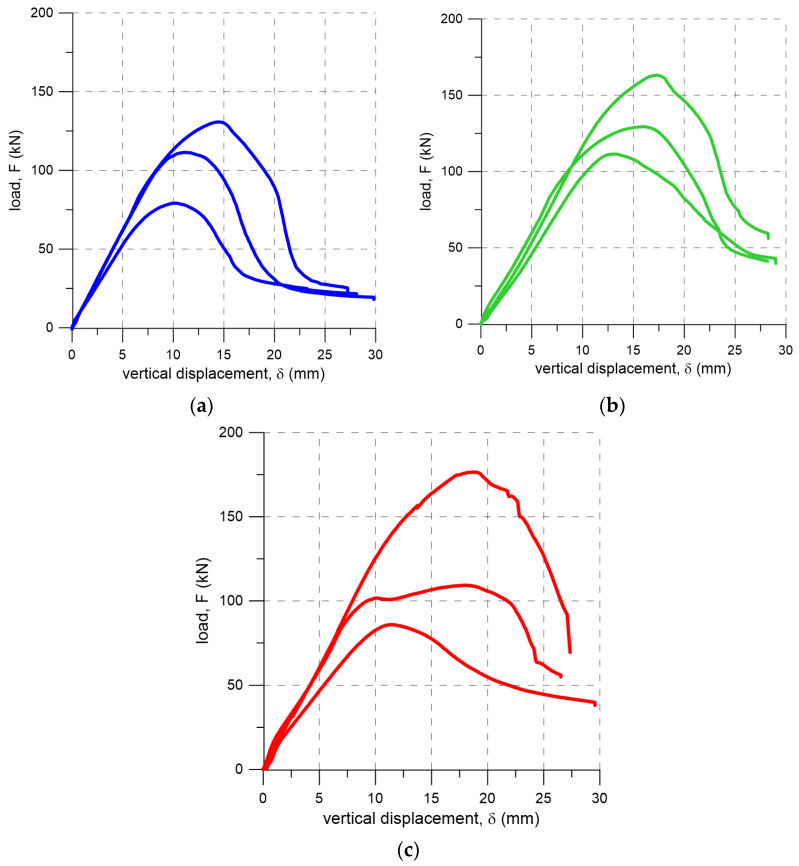
Load–vertical displacement curves from shear tests performed under three precompression levels: (**a**) 0.2 N/mm^2^; (**b**) 0.6 N/mm^2^; (**c**) 1.0 N/mm^2^.

For each precompression level, the average peak load (F_max,av_) and the corresponding coefficient of variation (CV) are also reported. The vertical displacement versus load curves for each specimen are presented in Figure 8.

**Table 1:** Test results.

Notation	Precompr. (N/mm^2^)	Failure Mode	F_max_ (kN)	δ_Fmax_ (mm)	F_max,av_ (kN)
FRI-9-3	0.2	FM1/FM2 *	130.7	13.8	107.1
FRI-9-4	0.2	FM1	79.2	9.4	CV 24%
FRI-9-5	0.2	FM1/FM2 *	111.4	11.2	
FRI-9-1	0.6	FM1/FM2 *	129.4	15.8	134.7
FRI-9-6	0.6	FM1	163.1	17.3	CV 19%
FRI-9-7	0.6	FM1	111.5	13.0	
FRI-9-2	1.0	FM1	109.3	18.0	123.9
FRI-9-8	1.0	FM1/FM2 *	176.5	18.7	CV 38%
FRI-9-9	1.0	FM1	85.9	11.3	

CV—coefficient of variation; *—localized failure within the polyurethane layer.

Regardless of the precompression level, all specimens failed due to shear failure in the unit/flexible joint interface. In specimens FRI-9-1, FRI-9-3, FRI-9-5 and FRI-9-8, shear failure also developed locally within the polyurethane joint. The average maximum loads obtained in tests on specimens subjected to precompression levels of 0.2, 0.6, and 1.0 N/mm^2^ were 107.1 kN, 134.7 kN, and 123.9 kN, respectively. These loads were reached at average vertical displacements of 11.5 mm, 15.4 mm, and 16.0 mm.

**Figure 9:**
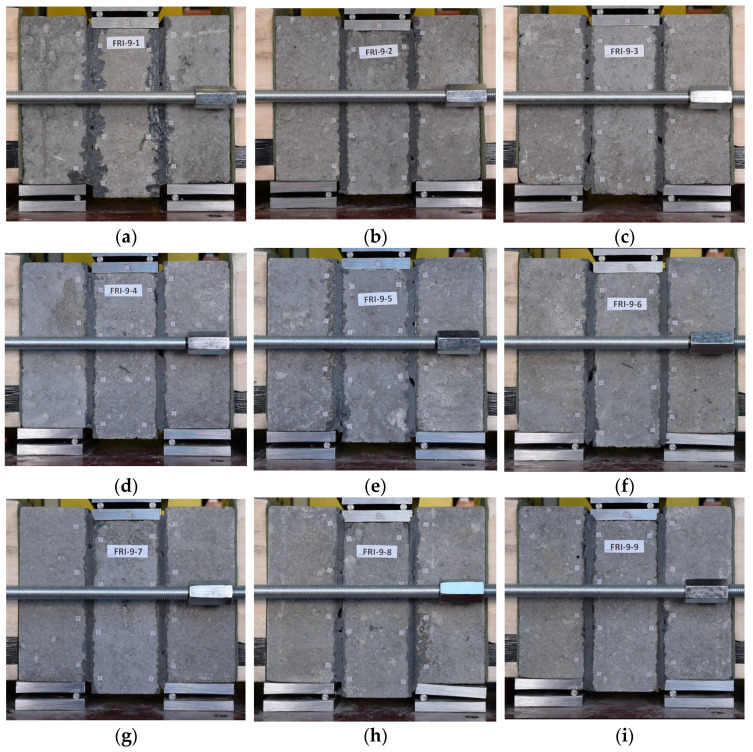
Failure modes obtained in the tests for specimen: (**a**) FRI-9-1; (**b**) FRI-9-2; (**c**) FRI-9-3; (**d**) FRI-9-4; (**e**) FRI-9-5; (**f**) FRI-9-6; (**g**) FRI-9-7; (**h**) FRI-9-8; (**i**) FRI-9-9.

Regardless of the precompression level, failure did not occur in a sudden manner and took place within the unit–flexible joint bond area (FM1). In several cases (see Table 1), failure within the polyurethane layer (FM2) was observed on limited area. The failure modes of all tested specimens are presented in Figure 9.

**Figure 10:**
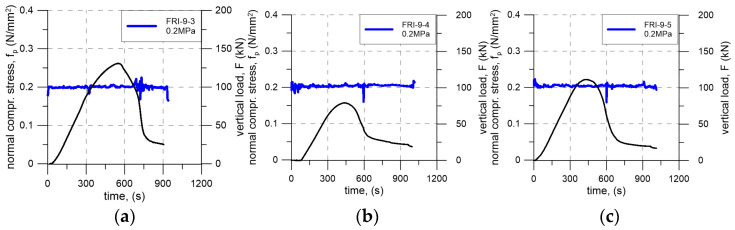

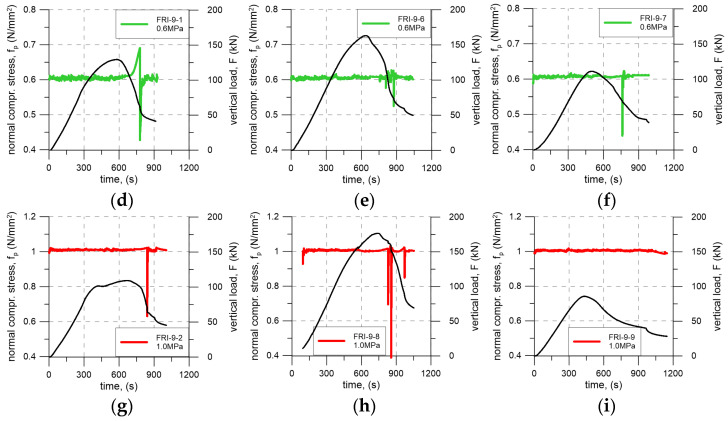
Time histories of normal compressive stress for specimen tested under various precompression levels: (**a**–**c**) 0.2 N/mm^2^; (**d**–**f**) 0.6 N/mm^2^; (**g**–**i**) 1.0 N/mm^2^. Blue, green, and red lines indicate normal compressive stresses, while the black line represents the vertical load–time history.

During the tests, the precompression level was continuously monitored and manually adjusted to maintain the target value. Variations in precompression resulted primarily from deformation of the tested element. As shown in Figure 10, which presents the time history of precompression for each specimen, the precompression level oscillated around the intended value. The largest deviations from the target level occurred after the maximum load had been reached. These oscillations were associated with the manual control procedure and with the mechanical response of the polyurethane adhesive layer. At large shear deformations, local loss of continuity within the adhesive layer led to geometric instabilities, manifested as wrinkling and interlayer roll-over (folding) of the polyurethane layer. However, these effects did not affect the measured shear strength results, as the consistency of the test conditions was preserved during the critical loading phase.

## 4. Discussion

**Figure 11:**
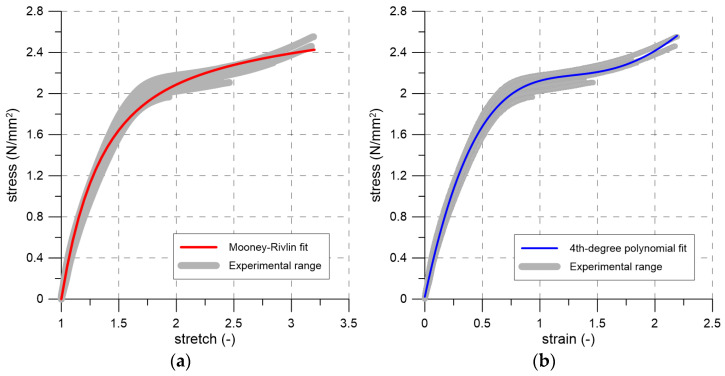
(**a**) Stress–stretch envelope from axial tensile tests of PM-type polyurethane, together with the Mooney–Rivlin fit; (**b**) Stress–strain envelope from axial tensile tests of PM-type polyurethane, together with the 4th-degree polynomial fit.

The tensile tests performed on PM-type polyurethane dumb-bell specimens were used to calibrate the material model for a computational analyses. A commonly used constitutive description for hyperelastic materials is the Mooney–Rivlin model. Based on the experimentally obtained stress–stretch curves, the two primary model parameters were identified as C_10_ = 0.0488 N/mm^2^ and C_01_ = 1.095 N/mm^2^. The uniaxial form of the Mooney–Rivlin model is shown in Figure 11a together with the experimental stress–stretch envelope. For this calibration, a coefficient of determination of R^2^ = 0.992 was obtained. As illustrated in the figure, the proposed model does not accurately reproduce the material behavior at larger deformations (stretch values exceeding approximately 2.5). Therefore, in Figure 11b, the experimental data (stress and strain) were additionally approximated using a fourth-degree polynomial with the following coefficients (in MPa): 0.0231, 5.28, −4.82, 1.89, −0.249, corresponding to the zero- through fourth-order terms, respectively. In this case, the coefficient of determination improved to R^2^ = 0.996, indicating that the polynomial representation provides a better overall fit to the observed material response.

It is worth noting that the polyurethane material used demonstrates the ability to accommodate large deformations, exceeding 200%, with a moderate tensile strength of 2.27 N/mm^2^ and a Young’s modulus of approximately 6 N/mm^2^. Traditionally, when filling voids, spaces, or cracks in buildings, mineral-based materials (such as mortars and injections) or epoxy-based materials (e.g., injections) are employed. However, these materials are brittle. Mineral materials exhibit high compressive strength, up to 40 N/mm^2^, but have low tensile strength, typically around 10% of their compressive strength. Failure stress occur at strain around 0.02% [35,36,37,38]. Epoxy materials are characterized by relatively high tensile strength (ranging from 40 N/mm^2^ to 100 N/mm^2^) and elongation at break between 1% and 5% [39,40,41]. The Young’s modulus of mineral mortars, depending on their composition, ranges from 1000 N/mm^2^ for lime-based mortars to 8000 N/mm^2^ for cement-based mortars. For epoxy resins, the modulus typically ranges from 1000 N/mm^2^ to 4000 N/mm^2^. The use of polyurethane PM-type in joints allows for increased ductility, enabling the transfer of larger deformations compared to traditionally used materials. This, combined with the excellent damping properties of polyurethane [42,43], makes it particularly suitable for applications in seismic areas.

**Figure 12:**
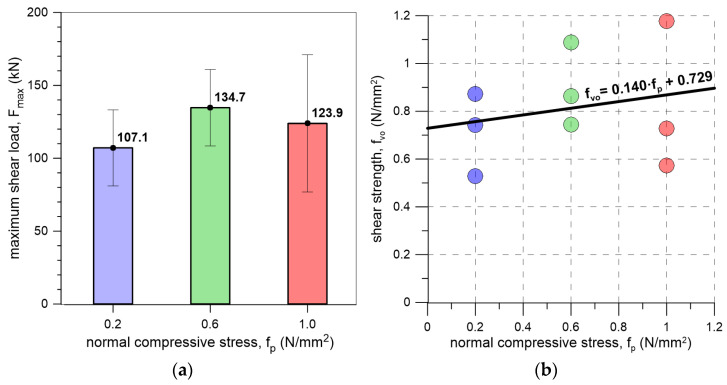
(**a**) Bar graph illustrating the mean and standard deviation (error bars) of maximum shear load obtained in the tests; (**b**) Scatter plot of shear strength versus precompression level for tested specimens, including the linear regression fit.

Analysis of the shear test results (Table 1, Figure 12a) indicates that increasing the precompression level affects the ultimate shear load. For a precompressive stress of 0.2 MPa, the lowest maximum shear load was observed (107.1 kN). Specimens subjected to a precompressive stress of 0.6 MPa failed at higher load levels, with an average value of 134.7 kN. Further increasing the precompressive stress led to the highest recorded shear load of 176.5 kN for sample FRI-9-8. It should be noted, however, that the scatter of the results (coefficient of variation) was relatively high across all test series, reaching up to 38% for the series with a precompressive stress of 1.0 MPa.

All specimens, regardless of the precompression level, failed due to shear failure in the unit–flexible joint bond area (FM1 failure mode) without any damage to the masonry units, indicating that the adhesive bond between the concrete block and the polyurethane layer governs the overall shear capacity of the connection. Under the tested conditions, the bond strength is therefore the limiting factor. This highlights the critical importance of careful surface preparation, including thorough cleaning, degreasing, priming, and maintaining appropriate substrate moisture, as well as ensuring the correct time interval between primer application and the formation of the flexible joint. Optimizing these steps can improve adhesion, reduce the risk of interfacial failure, and enhance the durability of the joint. From a construction practice perspective, consistent attention to these procedures ensures effective load transfer across the interface, minimizes the risk of premature debonding, and allows designers to rely on the expected adhesive and cohesive performance of the polyurethane under both static and cyclic or seismic loading conditions.

In the studies available in the literature on the shear behavior of masonry elements joined with mineral mortar, the observed failure mode depends on the magnitude of the compressive stress, the strength of the masonry units and mortar, and the thickness of the mortar joint. When the level of precompression was relatively low, shear failure was observed in the mortar, at the unit/mortar bond area, or within the unit. For higher levels of compressive stress, failure occurred through crushing and/or splitting in the units [30,31,44,45,46,47,48]. If an additional layer of damp-proof course was placed in the joints, for low precompression levels, the typical failure mode was shear failure in the mortar/damp-proof course interface, while at higher precompression levels, mixed failure modes—shear/crushing in the units—were observed [23,49,50].

Based on the [22] standard, the initial shear strength of masonry made with concrete blocks joined by a flexible joint (triplet specimens) was determined. For this purpose, the shear strength values obtained from individual tests were plotted against the corresponding precompressive stress, and a linear regression of the data points was performed, allowing the determination of the initial shear strength at zero precompression (Figure 12b). If the connection were assumed to behave according to friction model, the internal friction angle could also be derived. In this case, the initial shear strength (*f_vko_*) was found to be 0.729 N/mm^2^, with a corresponding friction angle of 8° and a friction coefficient (*µ*) of 0.14. These values can be used in the computational analyses of flexible joints in masonry structures. In the studies of solid blocks joined with mineral mortar available in the literature, typical values for the friction coefficient range from µ = 0.3 to 0.8, and the initial shear strength is reported to be f_vko_ = 0.05–0.4 N/mm^2^ [30,45,46,48,51,52,53]. The relatively low friction coefficient and high initial shear strength of blocks with flexible joint, compared to typical masonry–mortar interfaces indicate that the shear resistance of the polyurethane joint is governed primarily by adhesion and the cohesive response of the polyurethane, rather than by frictional interlocking. In practice, this means that load transfer is largely controlled by bond quality and material integrity, making the joint less sensitive to normal stress but more dependent on the durability of adhesion. Under seismic or cyclic loading, this behavior can be advantageous due to the energy dissipation and deformation capacity of the polyurethane; however, it also emphasizes the importance of maintaining adhesion, as friction alone provides only limited resistance.

**Figure 13:**
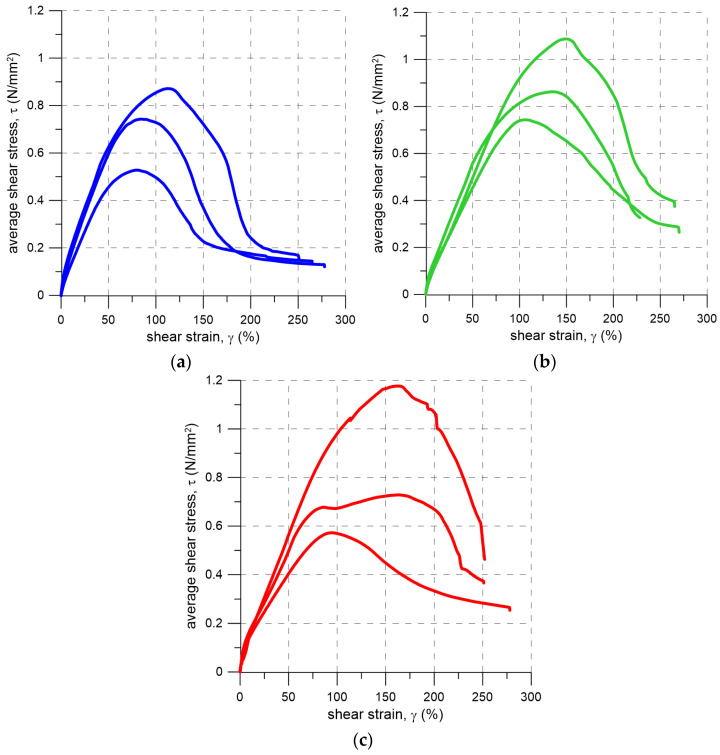
Average shear stress–shear strain curves from shear tests performed under three precompression levels: (**a**) 0.2 N/mm^2^; (**b**) 0.6 N/mm^2^; (**c**) 1.0 N/mm^2^.

During the tests, the Digital Image Correlation (DIC) method was used to monitor the relative displacement of the concrete blocks. Four measurement points were applied on each side of both joints of the specimens. Based on the obtained data, shear stress–shear strain curves were plotted to describe the behavior of the flexible joint under shear loading (Figure 13). The results indicate that the flexible joint made of PM-type polyurethane exhibits linear behavior up to a shear strain of approximately 50%. Beyond this deformation, a clearly nonlinear response is observed. The curves also demonstrate post-peak behavior. It is noteworthy that such a flexible joint, when subjected to precompressive stress, is capable of sustaining significant shear stresses even after debonding between the polyurethane and the concrete. Flexible joint with a low precompressive stress of 0.2 N/mm^2^ retains a shear strength of up to 0.15 N/mm^2^ at shear strains exceeding 200% (Figure 13a). The curves suggest a frictional nature of the interface between the flexible joint and the structural elements following debonding between the joint and the concrete. The possibility of achieving such large shear strains in the case of the flexible joint results from the high deformability of the PM-type polyurethane; in contrast, based on the literature, mineral mortars exhibit a limited deformation capacity and reach their shear strength at relatively low shear strains, typically around 5% [25,45,54,55,56].

## 5. Conclusions

This paper presents the outcomes of initial shear strength tests conducted on specimens made with concrete blocks connected with flexible joints. The joints were made of polyurethane PM-type. The primary conclusions drawn from the study are as follows:

- The Mooney–Rivlin model calibrated using tensile test data (C_10_ = 0.0488 N/mm^2^, C_01_ = 1.095 N/mm^2^) provided a good approximation of the behavior of the polyurethane (R^2^ = 0.992), although its accuracy decreased at larger deformations. A fourth-degree polynomial yielded an improved fit (R^2^ = 0.996), indicating that this representation more reliably captures the material response over the full strain range.
- Increasing the level of precompression resulted in higher ultimate shear loads, rising from 107.1 kN at 0.2 MPa to 176.5 kN at 1.0 MPa. However, the results exhibited considerable scatter, with coefficients of variation reaching up to 38%, indicating notable variability in the shear capacity across test series.
- All specimens failed at the unit–flexible joint interface, with no damage to the masonry units. This underscores the need for careful preparation of the faces of jointed elements prior to polyurethane application.
- A linear regression of shear strength versus precompressive stress, following EN 1052-3, yielded an initial shear strength of 0.729 N/mm^2^, corresponding to a friction angle of 8° and a friction coefficient of 0.14. These parameters provide a basis for computational analyses of flexible joints in masonry structures.
- DIC measurements of block displacements were used to plot shear stress–shear strain curves, characterizing the flexible joint’s behavior under shear loading.
- The tests were conducted for a single type of polyurethane and a single type of masonry unit, so the results have limited general applicability. Further studies are planned to investigate other polyurethanes and masonry units.

## Data Availability

The original contributions presented in this study are included in the article. Further inquiries can be directed to the corresponding author.
